# Caring for the caregiver: Why policy must shift from addressing needs to enabling caregivers to flourish

**DOI:** 10.3389/fpubh.2022.997981

**Published:** 2022-10-19

**Authors:** Brian Beach, Louise Bélanger-Hardy, Susana Harding, Monica Rodrigues Perracini, Linda Garcia, Ishika Tripathi, Margaret Gillis, Briony Dow

**Affiliations:** ^1^Research Department of Epidemiology and Public Health, University College London, London, United Kingdom; ^2^LIFE Research Institute, University of Ottawa, Ottawa, ON, Canada; ^3^International Longevity Centre – Singapore, Singapore, Singapore; ^4^International Longevity Centre – Brazil, Rio de Janeiro, Brazil; ^5^International Longevity Centre – Canada, Ottawa, ON, Canada; ^6^National Ageing Research Institute, Parkville, VIC, Australia

**Keywords:** unpaid caregivers, informal care, policy, global aging, flourishing, positive psychology

## Abstract

Policies supporting caregivers (“caregiver policies”) are limited in the extent to which they meet the needs of those who care for others. Where policies do exist, they focus on relieving the burdens associated with caring or the needs of the person they care for, rather than consider the holistic needs of the caregiver that would enable them to flourish. We argue that the established approach to caregiver policies reflects a policy failure, requiring a reassessment of current practice related to caregiver support. Often, caregiver policies target the care recipient rather than the caregiver's needs. Through a consultative exercise, we identified five areas of need that existing caregiver policies touch upon. Yet current approaches remain piecemeal and inadequate in a global context. Caregiver policies should not just relieve burden to the extent that caregivers can continue in the role, but they should support caregivers to flourish, and future work may benefit from drawing on related frameworks from positive psychology, such as the PERMA™ model; this is important for both policymakers and researchers.

## Introduction

Policies supporting unpaid caregivers (“caregiver policies”) are limited in the extent to which they meet the needs of those who care for others outside formal care systems. Where policies do exist, they focus on relieving the burdens associated with caring or the needs of the person they care for. They fail to fully support caregivers, thus impeding their ability to flourish or achieve wellbeing through positive emotion and positive functioning, despite the responsibilities they have assumed. Similarly, research on caregivers has largely adopted the same perspective.

We argue that the established approach to caregiver policies reflects a policy failure, requiring a reassessment of current practice related to caregiver support. As in many other areas of policymaking, a more integrated and holistic approach to policy development would be advantageous, particularly in a global context. To achieve an effective shift in caregiver policies, the narrative must move beyond one of burden to one that enables individuals for whom caregiving plays a major role to flourish in their overall lives.

## The importance of unpaid caregivers is growing around the world

Unpaid caregiving is a substantial component of modern societies' approach to those in need. This phenomenon is bound to increase during the next decades. Indeed, countries around the world are experiencing changes to the age structure of their populations in the context of improved longevity and lower fertility, with increasing proportions of the population being aged 65+ (1). At the same time, many people will develop chronic illness or disability that will mean they need care and support services (2). Globally, unpaid caregivers—often partners, adult children, or close friends—provide the vast majority of care and support received by older adults (3). While there is diversity across the world with respect to the design and delivery of formal long-term care (LTC) provision, unpaid caregivers are always crucial to support the needs of older people (4). Even in countries where there are well-developed LTC services, such as Australia, 80% of the care for older people is provided by family members (5).

These caregivers are not doing well. While some may experience positive emotions related to the caring trajectory (6), they are often exposed to time-consuming, exhausting, and stressful care conditions (3, 7). The substantial negative impact of providing unpaid care upon the caregiver's own physical and mental health, employment prospects, and social participation has been extensively recognized (3, 8–12).

The fact that these trends will characterize societies across the coming decades is not a new observation. Policymakers, scholars, and advocacy organizations focused on caregivers have long highlighted the growing and anticipated challenges brought about by these demographic changes. There has been progress in some countries toward recognizing caregivers and implementing policies to support them, but challenges remain both in terms of ensuring consistency across contexts and in shaping positive outcomes for caregivers. Without action, insufficient caregiver policies may result in an alarming scenario in years to come, particularly in lower- and middle-income countries where the majority of older people will be living (13).

## Current policies fail to address the needs of caregivers

To understand the extent to which caregiver policies successfully address the needs of caregivers, a coherent framework for capturing the diversity of policies across cultural, economic, political, and social contexts would be useful. Currently, however, there is little global consistency in policy and programs to support caregivers and no common framework. This is not to say that efforts have not been made. For example, in its 2021 report, *Global State of Caring*, the International Alliance of Carer Organizations sets out key initiatives across six priority areas that are universal to caregivers: recognition; financial support; work and education; health and wellbeing; information and knowledge; and evidence-informed practices (14). While existing initiatives in these priority areas may assist the caregiver in the caring role, they mainly focus on enabling care provision rather than the wellbeing or flourishing of the caregiver.

One key challenge for caregiver policies is the extent to which existing policies and programs target the care recipient rather than unpaid caregivers themselves. Even where this support exists, the extent to which it effectively addresses unpaid caregivers' needs, let alone wellbeing, is unknown at best. The challenges around supporting unpaid caregivers through policy include:

- When policy is focused on the care recipient, unpaid caregivers are not explicitly recognized, leaving them with little agency and little attention to their own needs for support; moreover, they are frequently not involved in any decision-making processes related to policy development or in planning care.
- Even when supportive policies explicitly targeting unpaid caregivers are in place, there can be a lack of knowledge about them or around how to access and navigate them, particularly in the initial stages of the caregiving relationship.
- Means-testing is sometimes used to ensure resources are directed to those in the most financial need, but using means-testing for eligibility can exclude many people who would be eligible once care-related expenses are subtracted.
- Where good programs have been identified and created, scaling this up to the national level—particularly for large countries and those with extensive rural populations—will require resources and innovation.

Through a series of consultations, our team has reviewed existing literature and policies related to the provision of unpaid care. This process generated a consensus around a categorization of the needs of unpaid caregivers along with a ranking of their significance. Examples of existing policies that address these needs were collected to both illustrate what has been done and to provide signposting for future policy development based on existing practice. Importantly, this exercise has incorporated a global perspective, with participants from all populated continents and reflecting the diversity in socio-economic conditions that shape contemporary experiences of care and caregiving. Our discussions included perspectives from Australia, Brazil, Canada, Czechia, France, India, Israel, the Netherlands, Singapore, South Africa, the United Kingdom, and the United States.

Our categorization of unpaid caregiver needs is ranked from the “most addressed” to the least addressed. We maintain that it is challenging (if not impossible) to say that some needs are more important than others; our ranking instead reflects the extent to which there appear to be existing policies or policy attention given toward the categories of need we have identified.

Our ranked categories of needs are financial, emotional, resource-related, educational, and social. **Financial** needs are related to inadequate and/or uncertain income to face personal expenses or those related to providing care to the care-recipient. Financial support for caregivers can include direct income payments and supplements, credits toward tax, public assistance, other social security programs, and grants to cover care-related activities and costs.

**Emotional** needs arise from facing multiple and overwhelming tasks along with challenges faced in relationships with the care-recipient and or with other family members. Isolation and the arousal of doubts related to the ability to provide proper care may also jeopardize confidence and the sense of willingness to continue caring.

Providing care may also leave unpaid caregivers in need of broader supportive **resources** given directly to them. Such resource-based needs are related to mental health support, information on available community supports, accessible and appropriate facilities, external support and assistance that is most frequently associated with the provision of health and social care services to the home, support from family, friends and paid caregivers, respite care, and the provision of aids and appliances.

Along with such practical resources, caregivers need **educational** support so they can learn more about the condition of the care-recipient to help in solving specific caregiving concerns and accomplish daily tasks. Acute conditions can require caregivers to provide specific forms of care, such as managing pressure ulcers or catheters and helping with transfers and mobility. These needs may further require significant input from trained professionals.

Finally, caregivers in many countries are unrecognized and unsupported by society, resulting in invisibility and **social** isolation. There is a need to give them appropriate recognition to help raise awareness about their needs and their contribution to society. The demands on caregivers can often impact their social lives, requiring support to improve connections and contact with others, helping them to engage in social activities and a meaningful purpose in life.

In our different countries, we have been able to identify individual policies that seek to address these different areas of need separately, but we contend that such a piecemeal approach results in caregivers being let down, even when thinking strictly about needs. Reflecting on the framework of policy failure by McConnell (15), such minimal achievements in programs are insufficient to justify a declaration of success, given that the fundamental goals to enhance caregivers' lives appear unmet. While we look here across a set of policies and programs rather than at a specific one in-depth, this nonetheless suggests the need to realign policy objectives with outcomes that will generate a broad base of support among stakeholders.

## Enabling caregivers to flourish in and out of their roles

In the context of reflecting on policy success, we argue that unpaid caregivers should be considered **holistically** as people with a range of needs and personal ambitions—not just as people who can be relied on to deliver our broader social responsibility for the care of older adults. We argue that caregiver policies and programs should not just relieve burden to the extent that caregivers can continue in the role, but they should support caregivers to flourish. Other scholars have recently made similar arguments in the context of caregivers' wellbeing (16).

Let us take one example of policy approaches to caregivers that illustrates the importance of linking perspectives across areas. Among existing caregiver policies, an often-lauded approach entails efforts to enable the combination of paid work and unpaid caregiving. Governments remain keen to support full employment among working-age people and to facilitate extended working lives, yet the onset of care responsibilities is an important driver for early labor market exit into retirement and economic inactivity. Key measures to help caregivers remain in paid work include a legal right to request flexible working arrangements, a legal right to paid or unpaid leave to provide care, the protection of insurance benefits during these periods, and help for those returning to work.

Employment is a key element of social production and social participation; it can play a significant role for the caregiver in addressing financial, emotional, and social needs. Moreover, policies to help combine paid work and caregiving are welcomed by caregivers and their advocates alike—even if they are only part of the bigger picture. However, the onset of caregiving responsibilities also reflects the start of an often-prolonged period of unpaid work; even when caregivers exit the labor market, they can hardly be considered inactive.

This example, upon scrutiny, highlights both the benefit of adjusting the perspective on caregiver policies toward a more holistic one and the shortcomings of the current approaches in caregiver policies. These policies can provide tremendous benefits for caregivers across multiple areas of need while recognizing that caregivers are defined by more than their caregiving role. Where this kind of policy falls short is that it emphasizes the work alone as a productive activity, relegating the caregiving role to a secondary consideration: a worker who happens to provide care.

This example also suggests potential insights from applying the capability approach (17). This approach focuses on what people can do and the opportunities that enable them to pursue what they value. Recent scholarship has applied this framework in the context of work (18, 19); this is similar but not identical to the situation for unpaid caregiving. Existing theories often assume that work is chosen or that a specific job or working conditions can be changed, for example, when there is a mismatch in demands and resources, imbalance in efforts and rewards, or poor person-job fit (20–22). Unpaid caregiving, in contrast, generally arises due to need. Thus, if the caregiving situation cannot be changed, personal and contextual factors should be the target; policy can play an enabling support role.

We therefore assert that caregiver policies should be developed and/or expanded to take a person-centered approach to the caregiver. Otherwise, policies targeting certain elements of need, like financial support, may be more effective if designed in a universal way; after all, flexible working arrangements benefit workers of all ages in all kinds of settings, as the COVID-19 pandemic has shown. A strong approach to creating caregiver policies should focus on what it takes to help them thrive and flourish as individuals for whom caregiving plays a major role.

While there is no universally accepted definition of flourishing, it is conceptually closely linked to theories of positive psychology and wellness. The PERMA™ model is one model of flourishing that includes many elements of other models, such as those described by Hone et al. (23). The PERMA™ model covers social flourishing on a collective scale—e.g., relationships with others, finding greater purpose and meaning—as well as flourishing on an individual scale, such as through positive emotions and engagement. The PERMA™ theory of wellbeing highlights five key areas that contribute to human flourishing: positive emotion (“the pleasant life”); engagement (“activity as its own reward”); relationships; meaning; and accomplishment (pursuits for their own sake) (24, 25).

The PERMA™ model focuses on activities that individuals pursue for their own sake (24). Recent developments to assess wellbeing more holistically include indices useful for empirical studies of flourishing (26). The Flourish Index captures domains of happiness and life satisfaction, meaning and purpose, and close social relationships, along with domains specific to character and virtue and to physical and mental health: the latter being particularly relevant for studies of caregiving and aging (27). An extension to the index incorporates a sixth domain related to financial and material stability. Research has verified the validity and reliability of these indices across cultural settings, strengthening the case for future quantitative work to consider this model for flourishing (28, 29).

There has so far been little research on what would enable unpaid caregivers to flourish, as so much of the literature has focused on the burden of caregiving. However, the literature on satisfaction with care or more positive experiences of care has found a relationship between a better caregiving experience and greater intimacy and emotional closeness with the person cared for (30). Other research has found a link between better caregiving experiences and a sense of personal growth, including the development of patience and understanding, strength and resilience, and increased self-awareness and knowledge (31). In the context of ageing societies, models for flourishing must adopt a lifecourse perspective, recognizing that needs across various domains may change with age.

## Discussion

As we move through the twenty-first century, not only will greater numbers of older people require care in countries around the world, but greater numbers of people in mid- and later life will be supplying this unpaid care. The impact of increasing levels of caregiving extends beyond individuals and their own financial security, health, and wellbeing; they will impact societies on multiple levels, such as foregone tax revenue from employment. These impacts will demand different responses depending on national contexts, but all countries should recognize the potential economic consequences that could result from this shift.

Caregiver policies need a stronger and more positive approach to mitigate these impacts and proactively adapt to the changing trends. There is a need to strengthen and integrate existing resources, optimize existing laws and regulations, stimulate and promote inter-sectoral collaboration, and discuss the standards and regulation toward formal caregiving programs and courses. These areas should provide starting points for policymakers to work on new strategies to enhance the lives of caregivers and prepare for a future that will undoubtably be characterized by significant levels of unpaid care.

We have argued here that such steps should look beyond the existing burden-focused narrative around caregiving to consider the person at the center of caregiving, combined with considerations on how to enable caregivers to flourish as individuals and in their major roles of giving care to an older adult. We also call on fellow scholars and researchers to expand our evidence around caregiving to take more holistic perspectives that incorporate the concept of flourishing.

We know that care recipients depend on healthy, flourishing caregivers, yet research has predominately focused on the negative aspects of caregiving and not on the elements that allow caregivers to thrive. More research and evaluation on the policy options that support caregivers to flourish is required to ensure new and innovative policy approaches. The current system of piecemeal and inadequate polices does not support the vital role that individuals play in providing care to an ageing society. We need fresh research and fresh approaches to generate opportunities for innovation and new interventions to support our current and future caregivers.

The imperative for policymakers to adopt this shift is driven by much more than a moral obligation: there are significant economic costs of failing to do so, regardless of the current level of economic development in any given country. Research in England found the direct cost to the state from unpaid caregiving equates to between £172 and 252 million per year, with an additional £8.4–12.8 million per year for the National Health System to support caregivers; this contrasts with estimated total savings on professional homecare alone of £54–86 billion (32).

Unpaid caregiving saves governments money that they might otherwise have to spend on long-term care services. Caregivers who lack adequate support can easily fall out of the labor market, which can leave key sectors understaffed, reduce tax revenues to governments, increase demand for public income support, and stifle economic growth through lower consumer spending. Caregivers who face difficulty can also see their own health decline more rapidly, creating a cycle of increased demand on health and care services in the longer term. The experiences through the COVID-19 pandemic—in terms of burden due to no respite from care or the knock-on effects for supply chains due to absences from work to isolate with infected family—should make this imperative starkly clear.

Forewarned is forearmed, as the saying goes.

## Data availability statement

The original contributions presented in the study are included in the article/supplementary material, further inquiries can be directed to the corresponding author.

## Author contributions

BB, SH, MR, and BD developed text in earlier versions of the paper. MG and BD helped progress the text through an intermediate stage. LG and IT facilitated coordination across further iterations. BB, LB-H, and BD worked to produce a final draft. All authors reviewed and agreed on the final version.

## Funding

This work extends from an initiative by the International Longevity Centre (ILC) Global Alliance, which brought together experts from around the world to transform how we approach unpaid caregiving for older adults. Progress has been supported by the Global Ageing Research Partnership—project financed by the Polish National Agency for Academic Exchange (NAWA) PPI/APM/2018/1/00014/U/001—and the University of Ottawa LIFE Research Institute.

## Conflict of interest

The authors declare that the research was conducted in the absence of any commercial or financial relationships that could be construed as a potential conflict of interest.

## Publisher's note

All claims expressed in this article are solely those of the authors and do not necessarily represent those of their affiliated organizations, or those of the publisher, the editors and the reviewers. Any product that may be evaluated in this article, or claim that may be made by its manufacturer, is not guaranteed or endorsed by the publisher.
